# The Effect of Partial Sleep Deprivation and Time-on-Task on Young Drivers’ Subjective and Objective Sleepiness

**DOI:** 10.3390/ijerph20054003

**Published:** 2023-02-23

**Authors:** Nicola Cellini, Giovanni Bruno, Federico Orsini, Giulio Vidotto, Massimiliano Gastaldi, Riccardo Rossi, Mariaelena Tagliabue

**Affiliations:** 1 Department of General Psychology, University of Padova, 35131 Padova, Italy; 2 Mobility and Behavior Research Center—MoBe, University of Padova, 35131 Padova, Italy; 3 Department of Civil, Environmental, and Architectural Engineering, University of Padova, 35131 Padova, Italy

**Keywords:** drowsiness, fitness-to-drive, PERCLOS, SDLP, sleepiness

## Abstract

Despite sleepiness being considered one of the main factors contributing to road crashes, and even though extensive efforts have been made in the identification of techniques able to detect it, the assessment of fitness-to-drive regarding driving fatigue and sleepiness is still an open issue. In the literature on driver sleepiness, both vehicle-based measures and behavioral measures are used. Concerning the former, the one considered more reliable is the Standard Deviation of Lateral Position (SDLP) while the PERcent of eye CLOSure over a defined period of time (PERCLOS) seems to be the most informative behavioral measure. In the present study, using a within-subject design, we assessed the effect of a single night of partial sleep deprivation (PSD, less than 5 h sleeping time) compared to a control condition (full night of sleep, 8 h sleeping time) on SDLP and PERCLOS, in young adults driving in a dynamic car simulator. Results show that time-on-task and PSD affect both subjective and objective sleepiness measures. Moreover, our data confirm that both objective and subjective sleepiness increase through a monotonous driving scenario. Considering that SDLP and PERCLOS were often used separately in studies on driver sleepiness and fatigue detection, the present results have potential implications for fitness-to-drive assessment in that they provide useful information allowing to combine the advantages of the two measures for drowsiness detection while driving.

## 1. Introduction

Sleepiness is considered one of the most important factors contributing to road crashes [1,2]. Indeed, in the last decade, it has been reported that about half of adult drivers claim to have driven while drowsy and it has been estimated that about 9–20% of all motor vehicle crashes and 21% of fatal crashes are due to sleepiness [3,4,5]. Sleepiness is usually caused by insufficient sleep quality and/or quantity, as well as by the time of the day and homeostatic factors [6]. It is a common experience that after a night of fragmented sleep, the feeling of sleepiness and the urge to go to sleep the next day is increased. Driving after a total sleep deprivation period not only induces an increase in sleepiness and fatigue but also impairs driving performance [7,8,9,10], particularly in younger adults [11]. These effects seem to be present even after a single night of partial sleep deprivation (PSD), although the literature on partial sleep deprivation and sleepiness is scarce [12,13,14,15].

Sleepiness also depends on circadian factors. Indeed, sleepiness—and alertness—vary across the 24 h period, showing a critical decline and a marked increase in the pressure to fall asleep between 01:00 and 04:00 pm and between 04:00 am and 06:00 am [13]; this pressure increases after a PSD night. Interestingly, these time windows are overlapping with the two peaks of relative risk of car crashes (i.e., the ratio between the number of crashes and the volume of traffic), which are between 3:00 to 6:00 am, and between 1:30 to 4:00 pm [16]. Of note, although there is a long-lasting debate about sleepiness and alertness being reciprocal concepts or two independent mechanisms [17], empirical data indicate that sleepiness and alertness are highly correlated [18,19], and here we will refer to these terms as reciprocals.

Another important factor that may influence the relationship between sleepiness and driving behavior is fatigue. It has been shown that the characteristics of the driving environment may cause task-related (TR) fatigue in two different ways: active and passive fatigue see [20,21]. Active fatigue is the most frequent form of TR fatigue that drivers encounter. Some authors refer to active fatigue as mental overload in driving conditions, and passive fatigue as underload situations [22]. Examples of high-task demand situations include high-density traffic, poor visibility, or the need to complete an auxiliary or secondary task (i.e., searching for an address) in addition to the driving task. Passive fatigue is produced when a driver is mainly monitoring the driving environment over an extended period of time when most of the entire actual driving task is automated. Passive fatigue may occur when the driving task is predictable. Drivers may start to rely on mental schemas of the driving task which results in a reduction in effort exerted on the task. Underload is likely to occur when the roadway is monotonous and there is little traffic. The latter kind of TR fatigue is the one that is much more likely to lead to sleepiness, and experimental data show indeed a time-on-task effect, as prolonged time in monotonous scenarios can increase sleepiness and reduce alertness [23].

To increase the complexity of this scenario, it is worth mentioning that subjective and objective sleepiness are not the same concepts. Objective sleepiness is the tendency to fall asleep, characterized by several behavioral and physiological changes, such as the reduction of movement, the reduction of muscle tone, and the tendency of the eyes to close more often and for a longer time [17]. Several measures have been used to objectively monitor sleepiness while driving, which can be summarized into three main categories: vehicle-based measures, behavioral measures, and physiological measures [24]. Among the vehicle-based measures, the most reliable is considered the Standard Deviation of Lateral Position (SDLP), which is derived from the vehicle lateral position defined as the distance from the center of the vehicle to the central lane axis. In particular, positive and negative lateral positions represent a car’s position close, respectively, to the left and right edge line. A lateral position of zero indicates that the center of the car is precisely in the middle of the driving lane. The SDLP gives details about its variability on defined time–space intervals. Among the behavioral measures, the PERCLOS (i.e., PERcent of eye CLOSure over a defined period of time) is especially informative [24,25]. Subjective sleepiness, instead, is the craving to, and the individual perception of the need to, fall asleep. The most common measures employed to detect the level of sleepiness while driving are based on driver’s subjective estimations, and rating scales such as the Karolinska sleepiness scale (KSS, rating from 1—extremely alert to 10—extremely sleepy) are often used. Although literature indicates that ratings in the KSS higher than 5 are often associated with major lane departures, longer eye-blink duration, and drowsiness-related physiological signals [24], at the individual level, the association between objective and subjective sleepiness is very variable [26], and often there is a temporal lag between the peak of subjective and objective sleepiness [27].

While the literature seems to agree on a detrimental effect of total sleep deprivation on sleepiness while driving, only a paucity of studies has investigated the effect of a single night of PSD on sleepiness concurrently assessed by several proxy measures, such as the SDLP and the PERCLOS, as well as by subjective evaluation while driving. Here, we combined a within-subject design with the assessment of these indexes of sleepiness to assess whether and how a night of reduced sleep can impact different measures of sleepiness. Moreover, we assessed the interaction of time-on-task and partial sleep deprivation in increasing sleepiness over time. We expected that a single night of PSD would affect both objective and subjective sleepiness while driving. Specifically, compared to a full night of sleep, we expected to observe in our participants an increase in both subjectively reported sleepiness and behavioral indices of drowsiness, namely, SDLP and PERCLOS, which have been continuously monitored across 50 min of driving in a monotonous scenario. Moreover, we expected to observe an effect of time-on-task in both conditions (full night and partial PSD), with an increase in both subjective and behavioral sleepiness over the driving time. Lastly, based on previous studies using a similar protocol e.g., [28], we expected an interaction between time-on-task and sleeping condition for the behavioral measure of sleepiness, with a marked increase in both SDLP and PERCLOS over time in the PSD condition.

## 2. Methods

### 2.1. Participants

Twenty-seven active drivers (11 F, mean age was 24.93 ± 2.69 years) were recruited for the two-session driving simulation experiment. Five of these 27 participants were then discarded from the analysis due to technical issues (eye-tracking data of 4 participants and simulator data for another participant). The final sample was composed of 22 participants (6 F, mean age: 25.09 ± 2.78 years, range 21–31 years). All the participants held a driver’s license, on average from 8.27 years (SD = 2.66, range = 5–14), with 54.54% (*n* = 12) driving at least 5.000 km per year. On average, the sample consumes 0.9 cigarettes per day (SD = 1.71, range = 0–6), 7.27 unities of coffee per week (SD = 5.27, range = 0–16), and 4.59 unities of alcohol per week (SD = 3.77, range = 0–15). The study was approved by the Ethics Committee for the Psychological Research of the University of Padova (ID No.: C7ACFE5F4436C27AC5CA25ABD8F68129). Before participating in each experimental session, participants gave their formal written consent, which was completely voluntary. Participants received 25€ in cash for completing the whole study.

### 2.2. Driving Simulator

#### 2.2.1. Apparatus

**Figure 1:**
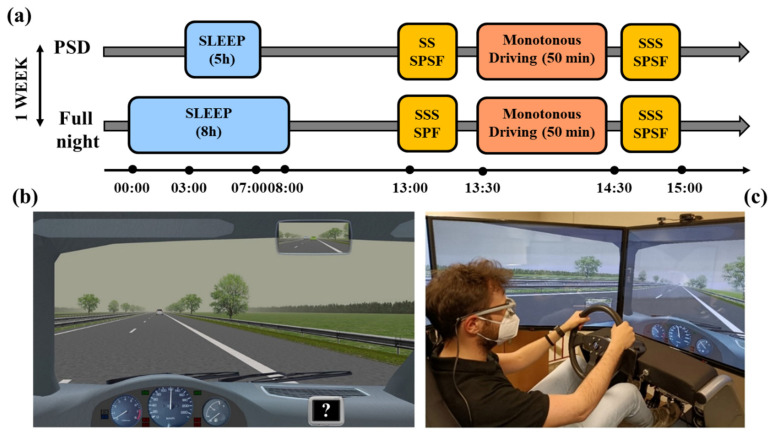
(**a**) Schematic representation of the experimental procedure. We used a within-subject design with all participants performing the study in two experimental conditions (full night of sleep and partial sleep deprivation) separated by one week. The order of the condition was counterbalanced across participants. (**b**) The driving scenario adopted for the experiments: Monotonous Environment. Note the question mark (?) on the dashboard monitor positioned on the lower-right part of the screen, which appeared every 9 min. (**c**) A participant driving in the dynamic simulator. PSD: partial sleep deprivation; SSS: Stanford Sleepiness Scale; SPF: Samn–Perelli Fatigue Scale.

A dynamic driving simulator with 2 degrees of freedom was used for the experiment. The simulator, which is located at the Mobility and Behavior Research Center (MoBe), and is produced by StSoftware^®^, has been previously validated [29,30] and used in several road safety studies [31,32,33,34]. As regards hardware, it consists of a cockpit with an adjustable seat, three pedals, manual gearbox, and a force-feedback steering wheel; the cockpit is surrounded by five 60-inch full-HD screens (330° by 45° field of view), and six speakers (three in the front, two in the rear, plus a subwoofer, Figure 1c). The simulation system collects thirty-one vehicle kinematic variables with a 50 Hz sampling frequency.

#### 2.2.2. Driving Scenario

We employed a monotonous scenario (Monotonous Environment, ME) of a highway environment, built in virtual reality using three-dimension rendering software. It was based on a dual-carriageway 164 km-long road segment. Each carriageway had two driving lanes (width 3.75 m) and a hard shoulder (width 3.00 m) with a virtual bank which created a tendency to deviate to the right, requiring drivers to make compensatory steering corrections. In the opposite direction, low traffic volume conditions were simulated to enhance the naturalism of the scenarios. The posted speed limit was 130 km/h.

In the ME, pairs of trees were regularly placed on both sides of the road, and facades of trees closed the line of vision at the horizon (Figure 1b). Daytime and cloudy weather conditions, which allowed good visibility (up to 500 m), were adopted. Wind or traffic disturbance effects on the lane were absent. Participants were asked to drive for 50 min in the center of the right-hand lane, as they would normally do in a natural setting, keeping a safe speed (choosing a speed suited to their normal driving style). They were not told exactly how long they were supposed to drive, they had to remove any wristwatch, and they were not given any information about how much time had passed.

#### 2.2.3. Standard Deviation of Lateral Position (SDLP)

From the driving simulator data, we computed the SDLP, which is widely considered the “gold standard” among the kinematic variables derived by the naturalistic driving observations, to assess the influence of sleepiness and drugs on driving behavior. It is considered a vehicle control measure, and its increase over the threshold of 2.5 cm allows to predict an increase of crash risk in drivers under the effect of alcohol, medications such as antidepressants or benzodiazepines, and sleep deprivation conditions [35]. In general, SDLP has been proven to be a real indicator of risk while driving under the effect of central nervous system drugs in a standardized on-road driving test [35,36] with normal traffic on public highways [37], being sensitive to the decrement in vigilance that is increasingly evident as the distance traveled increases [38]. In addition, Veldstra et al. [39] obtained comparable results on this variable in on-road and simulated driving after the administration of a cannabinoid medicine (dronabinol), in comparison with the placebo treatment. Moreover, measures of variability in lane position, in general, have been observed to be affected by sleep deprivation [35,40] and sleep-related disorders [41].

### 2.3. Eye-Tracker

Eye-gaze was recorded throughout the driving simulation using the SMI eye-tracking glasses 2 Wireless (SensoMotoric Instruments, Teltow, Germany), which is a non-invasive system designed similarly to a common pair of glasses and equipped with an HD scene camera (resolution 1280 × 960 p). Data were sampled at 60 Hz and were extracted using the BeGaze software (SensoMotoric Instruments, Teltow, Germany).

#### Percent of Eye Closure (PERCLOS)

From the eye-tracking data, we computed the PERCLOS-80 (P80) in single-minute bins. Assuming the 80% closure threshold, when the eyelid closure was below 20% of the detected maximum opening, the participants’ eyes were considered closed. The considered value was the percentage of closed eyes during each recording minute. To control for the impact of extreme values, data collected from minutes one and fifty were excluded from the analysis, and a 98% acceptance interval was considered in terms of P80. The PERCLOS has been shown to correlate with visual vigilance performance lapses [42] and it is widely used to monitor alertness in drivers [24,43].

### 2.4. Questionnaires and Subjective Sleepiness Measures

Before the first experimental session, we asked participants to fill out a set of questionnaires online, which provided us with information about the presence of sleep problems (Pittsburgh Sleep Quality Index; PSQI [44]), depressive symptomatology (Beck Depression Inventory-II, BDI-II [45]), anxiety level (State-Trait Anxiety Inventory, STAI-Y [46]), sleepiness level during the daytime (Epworth Sleepiness Scale, ESS) [47], and the circadian typology (Morningness-Eveningness Questionnaire reduced version, rMEQ [48]), as well as the demographics, which included also information about their driving license and their annual average driving distance. This information is reported in Supplementary Table S1.

During the experimental sessions, perceived sleepiness and fatigue were collected before and after the driving simulator using the Stanford Sleepiness Scale SSS [49] and the Samn–Perelli Fatigue Scale SPF [50], respectively. During the task, participants verbally reported every 9 min their level of sleepiness from 1 (“not at all”) to 10 (“extremely sleepy”), using a modified version of the KSS [14].

### 2.5. Procedure

We employed a within-subject design, with all participants performing the study in two conditions (full night of sleep and partial sleep deprivation) separated by 7 days (Figure 1a). Partial sleep deprivation was our experimental condition, since we manipulated (i.e., reduced) the time spent in bed, whereas the full night of sleep was considered our control condition. The experimental conditions were counterbalanced across subjects. In the full night of sleep condition, participants were asked to spend 8 h asleep the night before the experimental session. They were asked to go to sleep at 12:00 am ± 1 h and get out of bed at 8:00 am ± 1 h, for a minimum of 8 h in bed. On average, they reported a time in bed of about 8 and 34 min (SD = 46 min). In the partial sleep deprivation condition, they were asked to go to sleep at 2:00 am ± 1 h and get out of bed at 7:00 am ± 1 h, for a maximum of 5 h in bed. On average, they reported a time in bed of about 4 h and 59 min (SD = 10 min).

On the day of the experimental test, participants arrived at the lab between 01:00 and 03:00 pm and they filled out the SSS and the SPSF. Participants then completed the 50 min drive, after which they reported again their subjective level of sleepiness and fatigue. Then, participants were invited to sit in the simulator’s cockpit, where we calibrated the eye-tracker (SMI Tracking Glasses 2). Afterward, the participants drove for 50 min in the scenario described above. During the driving task, every 9 min, a question mark (?) appeared on a dashboard monitor positioned on the lower-right part of the screen (Figure 1a), and the participants had to verbally report their level of sleepiness from 1 (“not at all”) to 10 (“extremely sleepy”). The question mark remained on the screen for 5 s, and if the participants did not reply, we counted the event as a “missing” response. At the end of the driving phase, the eye-tracker was removed, and participants filled out the same questionnaire used before the driving; they were debriefed and then they could leave. All the participants were tested between 1:30 and 4:30 pm to increase the propensity to sleepiness.

### 2.6. Data Analysis

The statistical analysis was conducted in the R environment (version 4.1.1 [51]). Initially, the gamma family distribution was set as a reference point for implementing two generalized mixed effects linear models (*glmer*, R package “lme4”) [52], with the participant as the random variable. The percentage of P80 in one minute (m1) and SDLP (m2) were set as dependent variables of the two models. Both of them included as fixed effects: Experimental Condition (Full Vs. Partial Sleep Deprivation Night), Experimental Time (five 10-min blocks), and Gender (Female, Male), as well as the interaction between Experimental Condition and Experimental Time. Subsequently, three mixed effects linear models (lmer) were also fitted considering the administered subjective measures. Sleepiness was assessed both while driving (5 data points per subject, range 1–10, m3) and prior to and after the two experimental sessions through the Stanford Sleepiness Scale (SSS, range 1–7, m4). Before and after each driving activity, individual evaluation of fatigue was also collected through the administration of the Samn–Perelli Fatigue Scale (SPF; m5). Predictors of the three lmer models were consistent with m1 and m2. Post hoc pairwise comparisons were computed when requested, using the R package “emmeans” [53]. Bonferroni’s correction was set as an adjustment method. The final datasets and further information about data analysis are retrievable in the Supplementary Material of the present manuscript.

## 3. Results

### 3.1. Behavioral Measures

**Figure 2:**
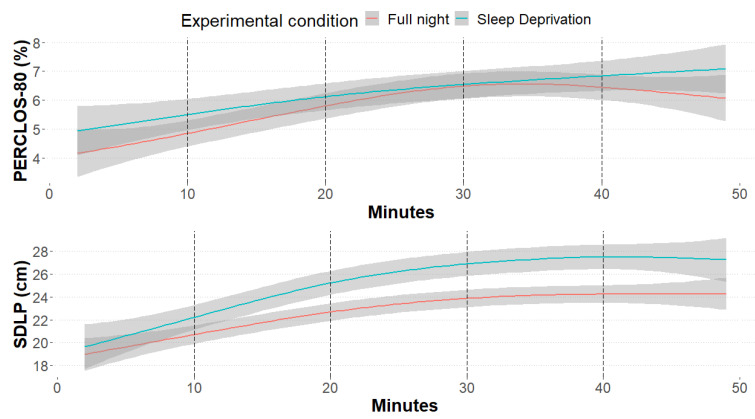
Smooth curves representing the percentage of eyelid closure below the 20% of the maximum openness (PERCLOS-80, **top**) and the standard deviation from lateral position (SDLP, **bottom**), divided by experimental condition (red: no sleep and 8 h sleep; blue: sleep deprivation and 5 h sleep).

PERCLOS-80 and SDLP trends throughout the driving session are described in Figure 2. In terms of P80, a main effect of the experimental condition was detected (χ^2^ _1_ = 22.59, *p* < 0.001), with higher scores in the case of sleep deprivation (6.23 v.s. 5.80%). P80 was observed to significantly grow over time (χ^2^ _4_ = 65.28, *p* < 0.001), particularly between the first 20 min and the rest of the driving session. Nonetheless, no significant difference between conditions throughout the experimental sessions was noted (χ^2^ _4_ = 4.13, *p* = 0.388), despite an interesting descriptive trend in the reduction of P80 in the Full Night condition while approaching the conclusion of the simulation. This behavioral trend was confirmed also by looking at SDLP scores, observing a stronger lateral mean deviation from the center of the lane in the sleep deprivation session (25.2 v.s. 22.7 cm; χ^2^ _1_ = 42.88, *p* < 0.001), as well as an increased deviation after 20 min driving (χ^2^ _4_ = 163.97, *p* < 0.001). Even in this case, no interaction between condition and driving time was detected (χ^2^ _4_ = 1.98, *p* = 0.738). Interestingly, drivers with less than 5.000 km travelled per year showed a drowsier eye activity than their counterparts (7.34 v.s. 4.87%, χ^2^ _1_ = 8.08, *p* = 0.004), but no differences were detected in terms of SDLP between the two conditions (Table 1). No significant gender differences were detected between the two measures.

### 3.2. Subjective Measures

**Figure 3:**
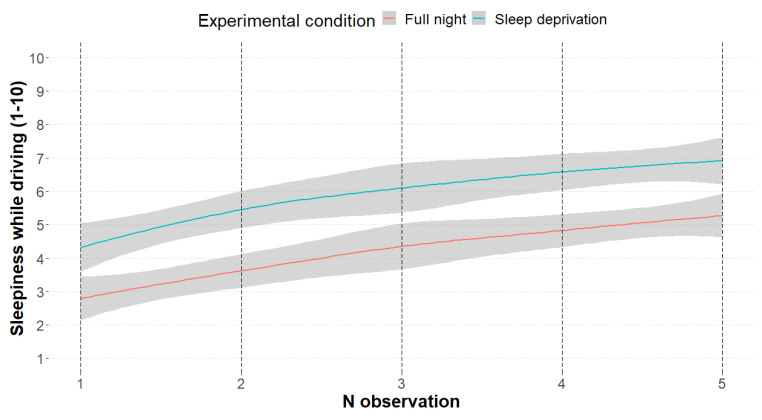
The smooth curves represent the subjective sleepiness scores among the 5 detection events (minutes: 9, 18, 27, 35, 45), divided by experimental condition (red: no sleep and 8 h sleep; blue: sleep deprivation and 5 h sleep).

**Figure 4:**
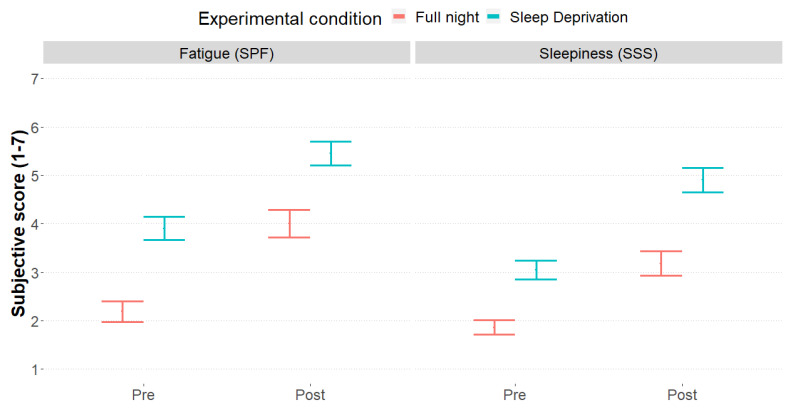
Mean and standard errors of fatigue scores (SPF, **left**) subjective sleepiness (SSS, **right**) and, divided by experimental phase (Pre, Post) and experimental condition (red: full sleep night with 8 h sleep; blue: sleep deprivation with 5 h sleep).

Sleepiness was evaluated also through the administration of two subjective measures, one administered during the driving sessions (*m3*), and the second presented before and after each experimental condition (full night, sleep deprivation; *m4*). As expected, results from both subjective measures were consistent with behavioral data. Looking at the streaming measure, sleepiness scores were higher in the sleep deprivation condition (5.92 v.s. 4.16; χ^2^ _1_ = 145.43, *p* < 0.001) and overall, as the session proceeded (χ^2^ _4_ = 154.77, *p* < 0.001; Figure 3). Seemingly, SSS scores described lower sleepiness scores after a full night of sleep (2.52 v.s. 3.98; χ^2^ _1_ = 65.65, *p* < 0.001), as well as predictable higher scores after the 50 min driving activity (4.02 v.s. 2.44; χ^2^ _1_ = 76.65, *p* < 0.001). Higher sleepiness among those who drove for less than 5.000 km per year was also detected from the scores of the subjective measures, confirming the behavioral trend (*m3*: χ^2^ _1_ = 6.20, *p* = 0.012; *m4*: χ^2^ _1_ = 5.20, *p* = 0.022). Predictably, also fatigue—detected through the SPF scale—followed sleepiness trends, confirming a great weariness in the sleep deprivation condition (χ^2^ _1_ = 91.60, *p* < 0.001), mainly after the driving simulation session (χ^2^ _1_ = 102.80, *p* < 0.001), and among less regular drivers (χ^2^ _1_ = 13.27, *p* < 0.001; Figure 4). Table 2 summarizes the descriptive scores of the three subjective measures considered.

## 4. Discussions

In the current study, we observed that a single night of partial sleep deprivation increases both subjective and objective sleepiness while driving compared to a full night of sleep. We also confirmed a time-on-task effect of driving on both objective and subjective sleepiness. However, we did not observe an interaction between sleep deprivation and time-on-task on either subjective or objective sleepiness.

A night of reduced sleep time impacted behavioral measures of sleepiness. Indeed, across the 50 min of driving, our participants showed an overall higher SDLP and PERCLOS after a PSD night compared to a full night of sleep. Moreover, we observed an increase in sleepiness after 20 min of driving, which continued growing until the end of the driving scenario, regardless of the sleeping condition the previous night. This increase was observed for both SDLP and PERCLOS. However, we did not observe a significant interaction between sleeping conditions and time-on-task for objective sleepiness.

Self-reported sleepiness showed a similar pattern. Indeed, a night of PSD was associated with a stronger feeling of sleepiness before the experimental task began, throughout the 50 min of driving, and after the task. However, the increase in subjective sleepiness throughout the task was similar between the conditions, indicating a lack of interaction between sleeping conditions and duration of the driving, and suggesting a specific time-on-task effect of sleepiness.

Our results are partially consistent with previous experimental studies on driving after PSD. For example, Zeller and colleagues [28] compared subjective, behavioral (SDLP), and physiological (EEG) indices of sleepiness in participants sleeping less than 5 h or more than 8 h while driving for 2 h in a simulator. In this between-subjects design, they reported a detrimental effect of PSD and time-on-task on self-report and EEG indices, but also an interaction between time-on-task and sleeping condition for SDLP, with increased SDLP in PSD participants during the last 10 min bin analyzed by the authors.

Another study [13] used a within-subject design, with participants in the PSD condition sleeping from 3:00 am to 7:00 am, and they assessed subjective, behavioral (SDLP and other vehicle-based measures), and physiological (EEG) indices of sleepiness in 90 min driving simulations. Testing participants, they observed a time-on-task effect on SDLP, EEG measures, and subjective sleepiness, while a PSD effect and an interaction between time-on-task and PSD were observed only for subjective sleepiness.

The effect of PSD on driving performance has been also reported by a recent study that tested professional (taxi drivers) and non-professional drivers in a simulator cab after a baseline test, one night of PSD (sleeping less than 4.5 h), and after two consecutive nights of PSD [15]. They reported driving impairment (e.g., increased SDLP) in the two PSD tests among participants regardless of their driving skills. Moreover, the same authors have previously shown [12] a reduced ability of drivers to perceive risks and respond faster in emergencies after either a single or two nights of PSD (sleeping less than 4.5 h).

Interestingly, our data are also consistent with the results of a large survey (*n* = 31,522) showing that short sleepers (i.e., participants that reported sleeping less than 6 h) report more often sleepiness behavior while driving (i.e., having nodded off or fallen asleep, even briefly), and this effect was more pronounced for very short sleepers ≤5 h [54]. This study also showed that the drowsy driving was reported independently of the participant’s perception of having gotten enough rest or sleep.

The present results should be considered in light of some limitations. First of all, our final sample size was small (N = 22), and this may have reduced the power of the study. We opted to follow a rule of thumb for the definition of the sample size, considering that several studies on drivers’ performance and driving behavior have developed important and solid results with less than 30 participants e.g., [7,13,19,55,56]. Importantly, we relied on a within-subject design, which is a widely recommended approach to increase statistical power [57] Second, our sample was mainly composed of young drivers (mean age: 25.09 ± 2.78 years; age range: 21–31 years); therefore, our results cannot be generalized to other populations. Further studies should try to reproduce the current results with a larger sample size and also consider different age populations. Moreover, as mentioned by Otmani and colleagues [13], it is important to remark that we obtained these results with a driving simulator, where participants were aware that driving errors would not affect their safety. Moreover, participants were not able to use real-life strategies to compensate for sleepiness, such as opening the windows or turning on the stereo. Nevertheless, driving simulators are useful to observe driving behaviors in conditions implying some amount of risk and the possibility to incur crashes and dangers. Moreover, driving simulators can be considered a valid tool, as was shown by Meuleners and Fraser [58], who demonstrated similarities in simulator performance and on-road behavior for some variables, such as attention to traffic lights and stop signals, space exploration, speed maintenance (especially at intersections), and mirror monitoring [59]. Thus, even though caution in interpreting study results from laboratory simulator tests is always recommended, similar effects on performance during simulated and on-road driving in testing, among others, fitness-to-drive under the effect of THC [39] and alcohol [60] have been observed, despite the somewhat lower sensitivity of the variables used to detect performance impairment in the former [60].

Another reason to use simulators, instead of on-road driving, is that laboratory conditions allow administering to participants the same standardized scenarios, which cannot be replicated in the same way on the road. For example, in on-road tests, traffic, sunlight, and road conditions (related to dirt, wetness, or the state of maintenance of the road markings) are not always the same and this might affect both the driver behaviors and the capability of in-vehicle instrumentation to correctly detect target kinematic variables [61].

Finally, considering risky situations such as those of driving under the influence of alcohol and drugs (medicinal or not), or in PSD conditions, on-road testing requires the presence of an instructor able to act to avoid crashes: this may affect the motivation of the drivers, modifying the phenomenon under observation [62]. Thus, in light of the pros and cons, in the present study, we decided that the use of the simulator represents a good compromise for practical, ethical, and safety constraints.

Some authors consider simulator fidelity as one of the key points which, in some way, can contribute to the generalizability of the results to the naturalistic conditions. Although a systematic review by Wynne et al. [59] showed that the relationship between validity and fidelity is not so clear and direct, the fidelity score of the simulator employed in the present study can be rated 4 out of 5, based on the dimension of visual, motion, and physical characteristics, given that it administers the road scenarios with a >270° FOV (horizontal) through five PC screens and includes vehicular controls identical to those of a real car, but without cab [for the criteria, see 59]. In addition, the simulator employed has been validated through a comparison with field observations, at least on the variable gap acceptance [30].

The present study has some practical implications. For instance, if drowsiness induced by a single night of PSD leads to an increase in the SDLP as we showed here, any infrastructural feature, such as different median separators that move the vehicle’s position away from the central trajectory (see, for instance [63]) could increase the risk of a run-off-the-road accident. Consequently, further studies should investigate the effectiveness of different types of median separation in drowsy conditions. Another possible extension of the present study could consider the effectiveness of warning systems signaling, for example, the proximity of a potential hazard, such as a sharp curve. Galante et al. [64], in a recent study, demonstrated that different delineation treatments can have a different impact on lane-keeping performance but all the treatments tested seemed to reduce SDLP. Thus, considering the detrimental effect of drowsiness on SDLP observed in the present study, it could be interesting to investigate whether some of the delineation treatments are better than others when the risk of drowsiness is also taken into account. The present results could also be used in public education (e.g., road safety campaigns), targeting young drivers who are at higher risk to have fatal road accidents. Indeed, young drivers are more susceptible to drowsiness while driving than older drivers, and are more involved in accidents due to falling asleep, fatigue, and drowsiness [65], as reported by the National Sleep Foundation [66]. Accordingly, May [67] explains this evidence because young drivers are more likely to drive when sleep-deprived, due to both school and extracurricular activities linked to their socialization habits. Moreover, in Italy, road accidents with drivers from 18 to 24 years old make up an impressive 64% of mortality of the drivers or the passengers, compared to the 44% of the general driving population [68]. These numbers highlight the need to study sleepiness in younger drivers.

## 5. Conclusions

In conclusion, our study showed that even a single night of partial sleep deprivation (i.e., sleeping less than 5 h) can affect objective and subjective sleepiness while driving. Moreover, our data confirm that both objective and subjective sleepiness increase throughout a monotonous driving scenario.

Finally, a general consideration of the potential practical applications of these results is required. Starting from the consideration that sleep disorders and sleep deprivation make drivers more vulnerable to crashes, Knott et al. [69], in their systematic review, showed that several studies have considered, among others, ocular measures (such as PERCLOS) and SDLP as, respectively, predictors and outcome of sleepiness. However, although the latter appears to be the most frequent vehicle metric in simulator studies, few studies considered these two variables jointly, and in some of them, the level of evidence in favor of the ability of ocular measures to predict adverse outcomes while driving is judged as insufficient.

Nevertheless, in a preliminary review of the techniques used to detect drowsiness while driving, Nasri et al. [70] carried out a comprehensive analysis of the pros and cons of three classes of measures, namely, physiological signals (EEG and ECG), facial features (including PERCLOS), and driving patterns (such as SDLP). The reviews’ conclusions were in favor of the experimental and practical use of facial features and driving measures, in light of their low level of intrusiveness. Considering also the highlighted limitations of facial feature recording and the fact that results are not so accurate in adverse light conditions, and the limitations of SDLP due to difficulties in trackside line detection depending on the conditions of the road, the results of the present study may contribute to investigations aimed at developing systems able to combine the two techniques, which can be implemented, in future applications, in the activity of real-time assessment of fitness-to-drive.

## Figures and Tables

**Table 1:** Mean and standard deviation (between brackets) of the percentage of eyelid closure below the 20% of the maximum openness (PERCLOS-80) and the standard deviation from lateral position (SDLP), divided by: experimental condition (full sleep night with 8 h sleep, sleep deprivation with 5 h sleep), experimental time (five 10 min blocks) and kilometers travelled per year (more or less than 5.000).

	PERCLOS-80 (%)	SDLP (cm)
Experimental condition		
Full night (8 h sleep)	5.80 (4.40)	22.7 (8.03)
Sleep deprivation (5 h sleep)	6.23 (5.82)	25.2 (10.9)
Experimental time		
2–10 min	4.71 (4.28)	20.1 (7.65)
11–20 min	5.64 (5.50)	23.0 (9.34)
21–30 min	6.52 (5.52)	25.0 (10.2)
31–40 min	6.61 (5.08)	25.6 (9.39)
41–49 min	6.51 (5.11)	25.9 (10.4)
Km per year		
Less than 5.000	7.34 (5.80)	23.8 (8.92)
More than 5.000	4.87 (4.25)	24.1 (10.3)

**Table 2:** Mean and standard deviation (between brackets) of the three subjective measures considered (range from 0 to 10, with 10 = maximum intensity), divided by: experimental condition (full sleep night with 8 h sleep; sleep deprivation with 5 h sleep), experimental time (only sleepiness while driving, five 10 min blocks), experimental phase (SSS and SPF, before and after the driving sessions), and kilometers travelled per year (more or less than 5.000).

	Sleepiness (While Driving)	Sleepiness (SSS, Pre-Post)	Fatigue (SPF, Pre-Post)
Experimental condition			
Full night (8 h sleep)	4.16 (1.75)	2.52 (1.17)	3.10 (1.46)
Sleep deprivation (5 h sleep)	5.92 (1.90)	3.98 (1.37)	4.66 (1.33)
Experimental time			
2–10 min	3.54 (1.45)	-	-
11–20 min	4.55 (1.84)	-	-
21–30 min	5.24 (1.95)	-	-
31–40 min	5.68 (1.94)	-	-
41–49 min	6.12 (1.90)	-	-
Experimental phase			
Pre	-	2.44 (0.98)	3.05 (1.34)
Post	-	4.02 (1.44)	4.71 (1.40)
Km per year			
Less than 5.000	5.61 (1.84)	3.55 (1.29)	4.51 (1.44)
More than 5.000	4.53 (2.05)	2.98 (1.55)	3.40 (1.55)

## Data Availability

The data that support the findings of this study are available from the corresponding author upon reasonable request. The study was not preregistered.

## Supplementary Materials

The following supporting information can be downloaded at: https://www.mdpi.com/article/10.3390/ijerph20054003/s1, Table S1: Demographics, driving, and psychological variables of the sample.

## References

[1] Gonçalves M., Amici R., Lucas R., Åkerstedt T., Cirignotta F., Horne J., Léger D., McNicholas W.T., Partinen M., Téran-Santos J. (2015). Sleepiness at the wheel across Europe: A survey of 19 countries. J. Sleep Res..

[2] Bioulac S., Franchi J.-A.M., Arnaud M., Sagaspe P., Moore N., Salvo F., Philip P. (2017). Risk of motor vehicle accidents related to sleepiness at the wheel: A systematic review and meta-analysis. Sleep.

[3] Owens J., Dingus T., Guo F., Fang Y., Perez M., McClafferty J., Tefft B. (2018). Prevalence of Drowsy-Driving Crashes: Estimates from a Large-Scale Naturalistic Driving Study. https://aaafoundation.org/prevalence-drowsy-driving-crashes-estimates-large-scale-naturalistic-driving-study/.

[4] Tefft B.C. (2014). Prevalence of Motor Vehicle Crashes Involving Drowsy Drivers, United States, 2009–2013.

[5] Klauer C., Dingus T.A., Neale V.L., Sudweeks J.D., Ramsey D.J. (2006). The Impact of Driver Inattention on Near-Crash/Crash Risk: An Analysis Using the 100-Car Naturalistic Driving Study Data.

[6] Dinges D.F. (2020). The nature of sleepiness: Causes, contexts, and consequences. Eating, Sleeping, and Sex.

[7] Jackson M.L., Croft R.J., Kennedy G., Owens K., Howard M.E. (2013). Cognitive components of simulated driving performance: Sleep loss effects and predictors. Accid. Anal. Prev..

[8] Marando I., Matthews R.W., Grosser L., Yates C., Banks S. (2022). The effect of time on task, sleep deprivation, and time of day on simulated driving performance. Sleep.

[9] Philip P., Taillard J., Micoulaud-Franchi J.-A. (2019). Sleep restriction, sleep hygiene, and driving safety: The importance of situational sleepiness. Sleep Med. Clin..

[10] Lowrie J., Brownlow H. (2020). The impact of sleep deprivation and alcohol on driving: A comparative study. BMC Public Health.

[11] Cai A.W., Manousakis J.E., Singh B., Kuo J., Jeppe K.J., Francis-Pester E., Shiferaw B., Beatty C.J., Rajaratnam S.M., Lenné M.G. (2021). On-road driving impairment following sleep deprivation differs according to age. Sci. Rep..

[12] Mahajan K., Velaga N.R. (2020). Effects of partial sleep deprivation on braking response of drivers in hazard scenarios. Accid. Anal. Prev..

[13] Otmani S., Pebayle T., Roge J., Muzet A. (2005). Effect of driving duration and partial sleep deprivation on subsequent alertness and performance of car drivers. Physiol. Behav..

[14] Åkerstedt T., Ingre M., Kecklund G., Anund A., Sandberg D., Wahde M., Philip P., Kronberg P. (2010). Reaction of sleepiness indicators to partial sleep deprivation, time of day and time on task in a driving simulator—The DROWSI project. J. Sleep Res..

[15] Mahajan K., Velaga N.R. (2022). Effects of partial sleep deprivation: A comparative assessment of young non-professional and professional taxi drivers. Transp. Res. Part F Traffic Psychol. Behav..

[16] Garbarino S., Lino N., Beelke M., Carli F.D., Ferrillo F. (2001). The contributing role of sleepiness in highway vehicle accidents. Sleep.

[17] Dement W.C., Carskadon M.A. (1982). Current perspectives on daytime sleepiness: The issues. Sleep.

[18] Kayumov L., Rotenberg V., Buttoo K., Auch C., Pandi-Perumal S., Shapiro C.M. (2000). Interrelationships between nocturnal sleep, daytime alertness, and sleepiness: Two types of alertness proposed. J. Neuropsychiatry Clin. Neurosci..

[19] Åkerstedt T., Folkard S. (1995). Validation of the S and C components of the three-process model of alertness regulation. Sleep.

[20] Rossi R., Gastaldi M., Gecchele G. (2011). Analysis of driver task-related fatigue using driving simulator experiments. Procedia Soc. Behav. Sci..

[21] Gastaldi M., Rossi R., Gecchele G. (2014). Effects of driver task-related fatigue on driving performance. Procedia Soc. Behav. Sci..

[22] May J.F., Baldwin C.L. (2009). Driver fatigue: The importance of identifying causal factors of fatigue when considering detection and countermeasure technologies. Transp. Res. Part F Traffic Psychol. Behav..

[23] Van Dongen H.P., Belenky G., Krueger J.M. (2011). A local, bottom-up perspective on sleep deprivation and neurobehavioral performance. Curr. Top. Med. Chem..

[24] Sahayadhas A., Sundaraj K., Murugappan M. (2012). Detecting driver drowsiness based on sensors: A review. Sensors.

[25] Dinges D.F., Grace R. (1998). PERCLOS: A Valid Psychophysiological Measure of Alertness as Assessed by Psychomotor Vigilance.

[26] Cai A.W., Manousakis J.E., Lo T.Y., Horne J.A., Howard M.E., Anderson C. (2021). I think I’m sleepy, therefore I am–Awareness of sleepiness while driving: A systematic review. Sleep Med. Rev..

[27] Manousakis J.E., Mann N., Jeppe K.J., Anderson C. (2021). Awareness of sleepiness: Temporal dynamics of subjective and objective sleepiness. Psychophysiology.

[28] Zeller R., Williamson A., Friswell R. (2020). The effect of sleep-need and time-on-task on driver fatigue. Transp. Res. Part F Traffic Psychol. Behav..

[29] Rossi R., Gastaldi M., Gecchele G., Biondi F., Mulatti C. (2014). Traffic-calming measures affecting perceived speed in approaching bends: On-field validated virtual environment. Transp. Res. Rec..

[30] Rossi R., Meneguzzer C., Orsini F., Gastaldi M. (2020). Gap-acceptance behavior at roundabouts: Validation of a driving simulator environment using field observations. Transp. Res. Proc..

[31] Orsini F., Gecchele G., Gastaldi M., Rossi R. (2019). Collision prediction in roundabouts: A comparative study of extreme value theory approaches. Transp. A: Transp. Sci..

[32] Orsini F., Zarantonello L., Costa R., Rossi R., Montagnese S. (2022). Driving simulator performance worsens after the Spring transition to Daylight Saving Time. Iscience.

[33] Rossi R., Orsini F., Tagliabue M., Di Stasi L.L., De Cet G., Gastaldi M. (2021). Evaluating the impact of real-time coaching programs on drivers overtaking cyclists. Transp. Res. Part F Traffic Psychol. Behav..

[34] Rubaltelli E., Manicardi D., Orsini F., Mulatti C., Rossi R., Lotto L. (2021). How to nudge drivers to reduce speed: The case of the left-digit effect. Transp. Res. Part F Traffic Psychol. Behav..

[35] Vinckenbosch F., Vermeeren A., Verster J., Ramaekers J., Vuurman E. (2020). Validating lane drifts as a predictive measure of drug or sleepiness induced driving impairment. Psychopharmacology.

[36] Ramaekers J. (2017). Drugs and driving research in medicinal drug development. Trends Pharmacol. Sci..

[37] Verster J.C., Roth T. (2012). Predicting psychopharmacological drug effects on actual driving performance (SDLP) from psychometric tests measuring driving-related skills. Psychopharmacology.

[38] Verster J.C., Roth T. (2013). Vigilance decrement during the on-the-road driving tests: The importance of time-on-task in psychopharmacological research. Accid. Anal. Prev..

[39] Veldstra J., Bosker W.M., De Waard D., Ramaekers J.G., Brookhuis K. (2015). Comparing treatment effects of oral THC on simulated and on-the-road driving performance: Testing the validity of driving simulator drug research. Psychopharmacology.

[40] Pizza F., Jaussent I., Lopez R., Pesenti C., Plazzi G., Drouot X., Leu-Semenescu S., Beziat S., Arnulf I., Dauvilliers Y. (2015). Car crashes and central disorders of hypersomnolence: A French study. PLoS ONE.

[41] Sagaspe P., Micoulaud-Franchi J.-A., Coste O., Leger D., Espié S., Davenne D., Lopez R., Dauvilliers Y., Philip P. (2019). Maintenance of Wakefulness Test, real and simulated driving in patients with narcolepsy/hypersomnia. Sleep Med..

[42] Wierwille W.W., Ellsworth L.A. (1994). Evaluation of driver drowsiness by trained raters. Accid. Anal. Prev..

[43] Mallis M.M., Dinges D.F. (2004). Monitoring alertness by eyelid closure. Handbook of human factors and ergonomics methods.

[44] Buysse D.J., Reynolds III C.F., Monk T.H., Berman S.R., Kupfer D.J. (1989). The Pittsburgh Sleep Quality Index: A new instrument for psychiatric practice and research. Psychiatry Res..

[45] Beck A.T., Steer R.A., Brown G.K. (1996). Manual for the Beck Depression Inventory-II.

[46] Spielberger C.D., Gorsuch R.L., Lushene R., Vagg P.R., Jacobs G.A. (1983). Manual for the State-Trait Anxiety Inventory.

[47] Johns M.W. (1991). A new method for measuring daytime sleepiness: The Epworth sleepiness scale. Sleep.

[48] Natale V., Esposito M.J., Martoni M., Fabbri M. (2006). Validity of the reduced version of the Morningness–Eveningness Questionnaire. Sleep Biol. Rhythms.

[49] Hoddes E., Zarcone V., Smythe H., Phillips R., Dement W. (1973). Quantification of sleepiness: A new approach. Psychophysiology.

[50] Samn S.W., Perelli L.P. (1982). Estimating Aircraft Fatigue: A technique with Application to Airline Operations.

[51] R Core Team (2021). R: A Language and Environment for Statistical Computing.

[52] Bates D., Mächler M., Bolker B., Walker S. (2014). Fitting linear mixed-effects models using lme4. arXiv.

[53] Lenth R., Lenth M.R. (2018). Package ‘lsmeans’. Am. Stat..

[54] Maia Q., Grandner M.A., Findley J., Gurubhagavatula I. (2013). Short and long sleep duration and risk of drowsy driving and the role of subjective sleep insufficiency. Accid. Anal. Prev..

[55] Morris D.M., Pilcher J.J., Switzer Iii F.S. (2015). Lane heading difference: An innovative model for drowsy driving detection using retrospective analysis around curves. Accid. Anal. Prev..

[56] Yang J.H., Mao Z.-H., Tijerina L., Pilutti T., Coughlin J.F., Feron E. (2009). Detection of driver fatigue caused by sleep deprivation. IEEE Trans. Syst. Man Cybern. Part A Syst. Hum..

[57] Maxwell S.E., Delaney H.D., Kelley K. (2017). Designing Experiments and Analyzing Data: A Model Comparison Perspective.

[58] Meuleners L., Fraser M. (2015). A validation study of driving errors using a driving simulator. Transp. Res. Part F Traffic Psychol. Behav..

[59] Wynne R.A., Beanland V., Salmon P.M. (2019). Systematic review of driving simulator validation studies. Saf. Sci..

[60] Jongen S., Vuurman E., Ramaekers J., Vermeeren A. (2016). The sensitivity of laboratory tests assessing driving related skills to dose-related impairment of alcohol: A literature review. Accid. Anal. Prev..

[61] Liu C.C., Hosking S.G., Lenné M.G. (2009). Predicting driver drowsiness using vehicle measures: Recent insights and future challenges. J. Safety Res..

[62] Helland A., Jenssen G.D., Lervåg L.-E., Westin A.A., Moen T., Sakshaug K., Lydersen S., Mørland J., Slørdal L. (2013). Comparison of driving simulator performance with real driving after alcohol intake: A randomised, single blind, placebo-controlled, cross-over trial. Accid. Anal. Prev..

[63] Calvi A., Cafiso S.D., D’Agostino C., Kieć M., Petrucci G. (2023). A driving simulator study to evaluate the effects of different types of median separation on driving behavior on 2 + 1 roads. Accid. Anal. Prev..

[64] Galante F., Mauriello F., Pernetti M., Rella Riccardi M., Montella A. (2022). Effects of traffic control devices on rural curve lateral position. Transp. Res. Rec..

[65] Shinar D. (2017). Traffic Safety and Human Behavior.

[66] National Sleep Foundation (2002). Adolescent Sleep Needs and Patterns: Research Report and Resource Guide.

[67] May J.F. (2011). Driver fatigue. Handbook of Traffic Psychology.

[68] DEKRA Automobil Rapporto Sulla Sicurezza Stradale. Mobilità Dei Giovani.

[69] Knott M., Classen S., Krasniuk S., Tippett M., Alvarez L. (2020). Insufficient sleep and fitness to drive in shift workers: A systematic literature review. Accid. Anal. Prev..

[70] Nasri I., Karrouchi M., Kassmi K., Messaoudi A. (2022). A Review of Driver Drowsiness Detection Systems: Techniques, Advantages and Limitations. arXiv.

