# Supplementary material for: The Effect of Partial Sleep Deprivation and Time-on-Task on Young Drivers’ Subjective and Objective Sleepiness
Source: Int J Environ Res Public Health. 2023 Feb 23;20(5):4003. doi: 10.3390/ijerph20054003 (PMC10001806; doi:10.3390/ijerph20054003)
Supplement: Supplementary file 1 [file ijerph-20-04003-s001.zip › ijerph-2182117-supplementary.pdf]

Supplementary Material

Table S1. Demographics, driving, and psychological variables of the sample.

**Age (years)**	25.41±2.73
**Gender (F/M)**	7/15 (31.8%/68.2%)
**PSQI**	5.68±2.72
**ESS**	6.36±3.22
**rMEQ**	14.18±2.99
**BDI-II**	4.55±3.46
**STAI-Y2**	7.41±4.90
**ESS**	6.36±3.22
**Average driving at year (km)**	
Less than 2500 km	2 (9.1%)
Between 2500 and 5000 km	8 (36.4%)
Between 5000 and 7500 km	6 (27.3%)
Between 7500 and 10000 km	1 (4.5%)
More than 10000 km	5 (22.7%)

Notes. Data are presented as Mean±Standard Deviation or frequency (percentage). PSQI: Pittsburgh Sleep Quality Index; BDI-II: Beck Depression Inventory-II, STAI-Y2: State-Trait Anxiety Inventory; ESS: Epworth Sleepiness Scale; rMEQ: Morningness-Eveningness Questionnaire reduced version, rMEQ.
